# Dynamic Behaviours of Monodisperse Double Emulsion Formation in a Tri-Axial Capillary Device

**DOI:** 10.3390/mi13111877

**Published:** 2022-10-31

**Authors:** Yuchen Dai, Haotian Cha, Nhat-Khuong Nguyen, Lingxi Ouyang, Fariba Galogahi, Ajeet Singh Yadav, Hongjie An, Jun Zhang, Chin Hong Ooi, Nam-Trung Nguyen

**Affiliations:** Queensland Micro-Nanotechnology Centre, Griffith University, Nathan, QLD 4111, Australia

**Keywords:** double emulsion, core–shell droplet, microfluidics, tri-axial capillary, computational fluid dynamics, dripping regime

## Abstract

We investigated experimentally, analytically, and numerically the formation process of double emulsion formations under a dripping regime in a tri-axial co-flow capillary device. The results show that mismatches of core and shell droplets under a given flow condition can be captured both experimentally and numerically. We propose a semi-analytical model using the match ratio between the pinch-off length of the shell droplet and the product of the core growth rate and its pinch-off time. The mismatch issue can be avoided if the match ratio is lower than unity. We considered a model with the wall effect to predict the size of the matched double emulsion. The model shows slight deviations with experimental data if the Reynolds number of the continuous phase is lower than 0.06 but asymptotically approaches good agreement if the Reynolds number increases from 0.06 to 0.14. The numerical simulation generally agrees with the experiments under various flow conditions.

## 1. Introduction

Double emulsions core–shell droplets are referred to as dispersed droplets containing a smaller one as the core. Double emulsions are highly desired for applications in drug delivery, food science, controlled release of substances, etc., because they can encapsulate a cargo in the core. Due to its significance, various designs for the formation of double emulsions have been extensively studied. Double emulsions are performed either simultaneously or successively and categorised accordingly into one-step and two-step processes. The former is simple and robust [1], while the latter is more efficient and controllable [2]. The one-step approach is the focus of our present work. One-step formation of double emulsions has been achieved with microfluidic devices. The working principle relies on the so-called Rayleigh-Plateau instability, where a liquid jet becomes unstable with its wavelength larger than the circumference. The jet consequently breaks up into segments with minimum surface area, forming a spherical shape [3,4]. Utada et al. [5] connected a glass cylindrical capillary with a square glass one and forced all liquid phases through an exit downstream. Varying the dimension of the outlet orifice can adjust the size of the droplets. The team also employed the breakup mechanism of a single emulsion to explain the size distribution of the double ones and defined an effective capillary number to classify the formation process as dripping and jetting regimes. Nie et al. [6] developed a co-flow microfluidic device in polyurethane elastomer to form double emulsions. The sizes of the core and the outer droplets were tuned with the flow rates of the three liquid phases. In general, double emulsion droplets with diameters from ten to hundreds of micrometres can be formed [1,5,7,8]. However, larger core–shell droplets with diameters ranging from hundreds of micrometres to several millimetres are also required for applications such as cell manipulations [9,10], macrocapsules [11], and microreactors [12]. Producing double emulsion droplets in this size range using typical microfluidic devices requires a significantly low flow rate of the outer phase, usually resulting in a drop accumulation issue because the gravity or buoyancy effect dominates the inertial drag. To prevent this problem, capillary devices were oriented vertically and scaled up to the so-called “millifluidics” [13]. Note that the term “microfluidics” is still valid for droplet smaller than a millimetre even if the device dimension is larger than that [14,15]. Shao et al. [15] experimentally investigated double emulsions in a dual-coaxial capillary with a 2.2 mm diameter tube as the outer channel. Adjusting the relative position of the ends of the inner and middle capillaries can switch the operation between two-step and one-step process. Their results demonstrated that the dynamic effect of the inner phase on the size of shell droplet is insignificant in the two-step set up, but drastically affecting it in the one-step process. Additionally, the team found that the mismatch in formation frequencies of core and shell droplets in the two-step process can be avoided with the one-step device. Schmit et al. [16] utilised a pendant drop method to produce millimetre-size double emulsions in a capillary tube with a 4 mm outer diameter. Their experimental results quantitatively demonstrated the effects of the inner and middle phase flow rates on the number as well as the size of the core in each droplet.

Though it is ultimately essential to obtain experimental data on double emulsions, numerical simulations are also of great importance for understanding and optimising the operation parameters for droplet formations. The velocity and pressure fields can be readily visualised, which are not always accessible with experimental methods. Zhou et al. [17] simulated double emulsions in a capillary device using a diffuse-interface framework. The calculation domain was simplified as a 2-dimensional axisymmetric geometry. The interfacial thickness and position were determined by a phase-field variable. By applying a similar method, Park and Anderson [18] successfully predicted the dripping and jetting regimes as well as their transition for the capillary device reported by Utada et al. [5]. Vu et al. [19] numerically investigated double emulsions in a capillary device using the front-tracking method proposed by Tryggvason et al. [20]. The results showed that the jetting mode can be promoted by increasing the Reynolds number (*Re*) and the Webber number (*We*). Fu et al. [21] established a model based on the ternary Lattice Boltzmann method to simulate the one-step process. Herrada et al. [22] carried out numerical simulations to validate their linear stability analysis on double emulsions in a capillary device. The team adopted the volume-of-fluid (VOF) method to simulate multiphases and interfaces. The effects of viscosity ratio, flow rate ratio, and interfacial tension ratio on the one-step process were also quantitatively demonstrated using the VOF method [23,24]. Azarmanesh et al. [25] simulated both one-step and two-step processes using the VOF method and found that the Capillary number (*Ca*) of the inner phase can significantly affect the outer droplet size in the two-step process, while the *Ca* of the outer phase plays a significant role in one-step counterpart. More recently, Yang et al. [26] numerically investigated the deformation behaviours of one-step double emulsions using VOF method. Examining the streamlines in the channel, the team discovered that the core moving forward relative to the shell was caused by the large vortex passing through the core–shell interface.

The dripping regime is well known to produce a monodisperse droplet size distribution while the jetting regime leads to a polydisperse counterpart. For most applications, a monodisperse emulsion is preferred. As a result, a simple force balance might be conducted to elucidate the formation mechanism and to predict the size distribution for various flow conditions. In general, the forces exerted on a double emulsion are the same as those on a single emulsion. These forces are kinetic, drag, interfacial tension, buoyancy, and Laplace pressure forces [27]. Note that the Laplace pressure was commonly neglected in symmetric and axisymmetric flows but has to be considered in a T-junction configuration due to the significant difference between the head and tail curvatures [28,29]. In addition, the drag force may have various forms while the others remain the same. The deviations were mainly caused by the different wall effect corrections when extending the Stokes drag of unbounded flows to confined counterparts [30,31]. Adjusting the continuous phase flow rate can tune the size of the droplets as its changes the drag force. Temperature can affect the surface tension and the viscosity and thus can be used to tune the droplet size [32,33]. Furthermore, to connect the model of the single droplet with the core–shell droplet, an equivalence of droplets volume ratio and input flow rates ratio was usually introduced [13,34]. Note that this hypothesis is valid if each droplet contains a single core.

Although efforts have been dedicated to double emulsion formation with microfluidics, most reported works were mainly based on either experiments or CFD simulation. Only a few can provide a relatively simple analytical model. Often, results from experiments, theory, and numerical simulation are inconsistent, preventing effective optimisation of devices and operation parameters for the generation of double emulsion. To our best knowledge, no past studies concurrently examined experiments, CFD simulations, and analytical models under the same condition. We propose in the present study a simple experimental setup, a vertically oriented tri-axial co-flow capillary device, to generate core–shell droplets in a one-step process. We investigate the mechanism of the double emulsion formation both experimentally and numerically. Furthermore, we develop and validate a simple but effective analytical model for predicting the droplet size distribution with the wall effect and according to various flow conditions.

## 2. Experimental Materials and Methods

### 2.1. Design and Fabrication of the Capillary Device

**Figure 1:**
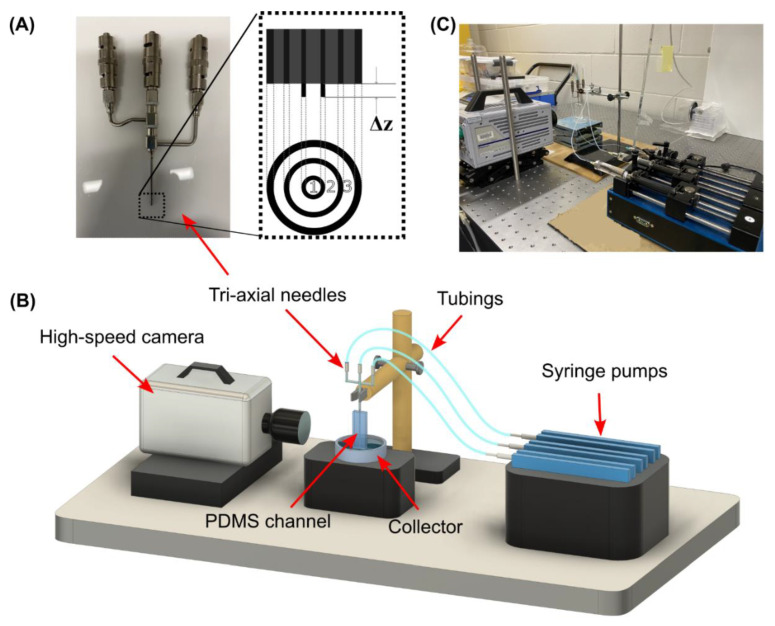
The capillary device used in this study: (**A**) the tri-axial needles; (**B**) the schematic diagram; (**C**) the photo of the experiment setup.

The tri-axial needles were purchased from Ramé-Hart instrument Co. (Succasunna, NJ, USA). The device structure and dimensions are detailed in Figure 1A and Table 1, with an inner needle tip extension Δ*z* = 0.1 mm. A straight circular channel was connected with the outlet of the needles. The outlet channel was made of poly-dimethylsiloxane (PDMS) moulded on a straight wire with the same diameter as that of the needles. The inlets of the needles are individually connected with three syringes via tubing. Figure 1B,C show the schematic diagram and the images of the experimental setup.

### 2.2. Materials

Fluorinated oil (HFE, Novec 7500 3 M, Merck, Darmstadt, Germany) was used as the core dispersed phase, whose density and dynamic viscosity are 1.61 g/mL and 1.31 mPa·s, respectively [8]. The shell dispersed phase was a polymer consisting of 0.06 g ethyl-4(dimethylamino) benzoate (Merck, Darmstadt, Germany), 0.05 g camphorquinone (Merck), and 10 g trimethylolpropane trimethacrylate (TMPTMA, Merck). The corresponding density and dynamic viscosity are 1.07 g/mL and 42 mPa·s, respectively [35]. The continuous phase was 80% glycerol dissolved in 20% DI water, with 0.1% Tween 20, and its density and dynamic viscosity are 1.21 g/mL and 75.42 mPa·s, respectively [36]. The laboratory temperature was maintained at 23 °C.

**Figure 2:**
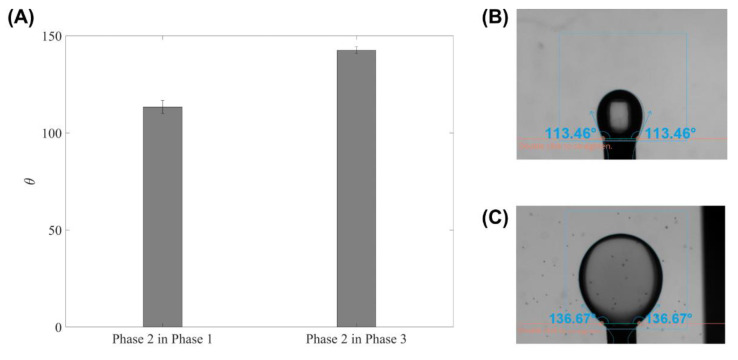
The interfacial tension coefficients measurement: (**A**) the mean contact angles (error bars represent the standard deviations and *n* = 7); (**B**) middle-phase reversed pendant drop in inner phase; (**C**) Middle phase reversed pendant drop in outer phase.

The interfacial tension $\sigma_{ij}$ between each pair of two liquid phases were measured using the reverse pendant drop method with an optical tensiometer (Theta Flex from Biolin Scientific, Gothenburg, Sweden). Figure 2A reports the mean contact angles of each pair of two phases, while Figure 2B,C show the images of the pendant drops. The interfacial tensions of the inner and outer interfaces were measured for 10 s for each case with 33 frames per second, and the measured time-averaged mean interfacial coefficients are 3.45 and 8.48 mN/m, respectively.

### 2.3. Experimental Setup and Data Analysis

The three phases were delivered into the capillary device at specific flow rates using syringe pumps (MNT-SPM-100, Welland, Austrlia). The formation and detachment of the droplets from the needle tips under various flow conditions were recorded using a high-speed camera (Photron FASTCAM SA3, San Diego, CA, USA, attached with a 25 mm F2.8 Ultra Macro 2.5-5.0X lens) at 250 frames per second. The open-source software ImageJ 1.53 m (National Institutes of Health USA, Bethesda, MD, USA) was adopted to analyse the captured videos.

## 3. Numerical Methodology

### 3.1. Governing Equations

All phases were modelled as incompressible fluids and laminar flows. The continuity and momentum equations are expressed as:

(1) $$\frac{\partial \rho }{\partial t}+\nabla \cdot \left(\rho \vec{v}\right)=0$$

(2) $$\frac{\partial \left(\rho \vec{v}\right)}{\partial t}+\nabla \cdot \left(\rho \vec{v}\vec{v}\right)=-\nabla p+\nabla \cdot \left[\mu \left(\nabla \vec{v}+\nabla {\vec{v}}^{T}\right)\right]+\rho \vec{g}+\vec{F}$$

where *ρ*, *t*, *v*, *p*, *µ*, g, and F denote density, time, velocity, pressure, dynamic viscosity, gravitational acceleration, and a source term, respectively. The Navier–Stokes equations above are related to the volume fraction of each phase $\alpha_{i}$. The density and viscosity in every single computational cell were calculated as follows:

(3) $$\left(\rho ,\mu \right)=\overset{3}{\underset{i=1}{\sum }}\alpha_{i}\left(\rho_{i},\mu_{i}\right)$$

The volume of fluid (VOF) method was adopted to model the fluid–fluid interfaces as:

(4) $$\frac{\partial \alpha_{i}}{\partial t}+\nabla \cdot \left(\alpha_{i}\vec{v}\right)=0$$

The sum of volume fractions of three phases within each computational cell follows the constraint:

(5) $$\overset{3}{\underset{i=1}{\sum }}\alpha_{i}=1$$

The continuous surface force (CSF) model developed by Brackbill et al. [37] was used to model the surface tension via the source term $\vec{F}$ in the momentum equation as:

(6) $$\vec{F}=\sum_{pairs\left(i,j\right),\;i<j}\sigma_{ij}\frac{\alpha_{i}\rho_{i}\kappa_{j}\nabla \alpha_{j}+\alpha_{j}\rho_{j}\kappa_{i}\nabla \alpha_{i}}{\frac{1}{2}\left(\rho_{i}+\rho_{j}\right)}$$

where κ stands for the interface curvature determined by:

(7) $$\kappa_{i}=\nabla \cdot {\hat{n}}_{i}$$

and $\hat{n}$ is defined as:

(8) $${\hat{n}}_{i}=\frac{\nabla \alpha_{i}}{\left|\nabla \alpha_{i}\right|}$$

### 3.2. Computational Domain and Boundary Conditions

**Figure 3:**
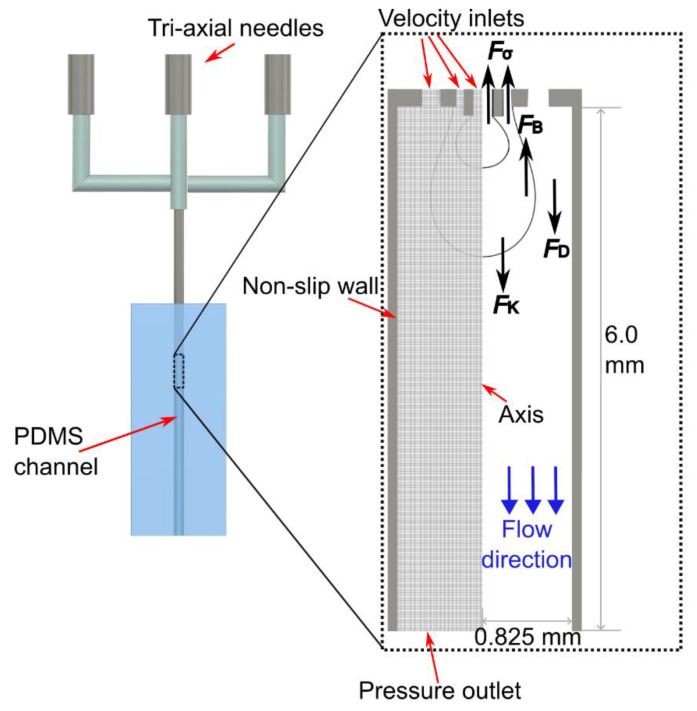
Schematic illustration of the computational domain, structural mesh, and forces exerting on a growing double emulsion droplet.

As the droplets are formed at the needle outlet, the corresponding circular cross-section channel downstream was selected to be the computational domain. The centreline was regarded as the axis due to the axisymmetric geometry. Boundary conditions at the entrance, exit, and walls were set to be velocity inlet, pressure outlet, and no-slip wall, respectively, Figure 3. The contact angles between each interface and the needle wall were determined based on experimental results in Figure 2B,C because the tri-axial needle and the hook needle used in the interfacial tension measurement are both made of stainless steel. The hydrodynamic entrance length for each needle inlet was estimated by $l_{ent,i}=0.0575Re_{i}D_{i}$ assuming a laminar pipe flow [38]. The corresponding order of magnitude in this study was calculated as $l_{ent,1}\sim O\left(1\;\mu m\right)$, $l_{ent,2}\sim O\left(0.1\;\mu m\right)$, $l_{ent,3}\sim O\left(10\;\mu m\right)$, respectively. To ensure each flow inlet is fully developed before reaching the expansion region, the inlet lengths for inner, middle, and outer needles in the simulation were fixed, respectively as 300, 200, and 200 µm, much longer than the hydrodynamic entrance lengths. Note that the inner needle inlet length was determined by considering its extension length. Structural meshes were generated, and a grid independence analysis was conducted with three cell sizes: 15 µm, 10 µm, and 5 µm with corresponding grid numbers of 23,652 (coarse), 51,785 (medium), and 204,428 (fine), respectively. The results showed that the medium and fine meshes, under the same input conditions, can model the interfaces with insignificant differences. Since the fine mesh drastically increases the computational resources, the medium one was selected for this study.

We performed simulations using the commercially available CFD software package ANSYS 2022. The SIMPLE algorithm was utilised for the pressure-velocity coupling. A PRESTO scheme was selected to calculate the exact value of the pressure term. We implemented the Geo-Reconstruct algorithm to acquire the interface interpolation. The governing equations were discretised by a second-order implicit scheme for the spatial terms and transient formulation. The time step was determined by the Courant–Fredrichs–Lewy number lower than 0.25.

## 4. Analytical Model

Both experiments and CFD simulations can capture the droplet formation in detail, but building empirical relations based on these methods usually requires relatively intensive case studies. Since we focus on the dripping regime, a simpler analytical model is more convenient for elucidating the droplet breakup mechanism and approximately predicting the droplet size. In the dripping regime, several forces are exerted on the growing droplets along the axial direction: viscous drag $F_{D}$ and kinetic $F_{K}$ forces pull them downstream while surface tension $F_{\sigma }$ and buoyancy $F_{B}$ forces act as opposite counterparts, Figure 3.

The assumptions for the analytical model are (i) the continuous flow is in the Stokes regime (*Re*_3_ << 1, where $Re_{3}=\frac{\rho_{3}r_{channel}{\bar{v}}_{3}}{\mu_{3}}$); (ii) the droplet formation is in the dripping regime (*Ca*_3_ < 1, where $Ca_{3}=\frac{\mu_{3}{\bar{v}}_{3}}{\sigma_{23}}$); (iii) the shape of droplets is spherical; (iv) laminar and incompressible flow are also presumed as per ansatzes in CFD simulations. The forces involved in the formation process are [13,27,39]:

(9) $$F_{K}=\rho_{1}Q_{1}v_{1}+\rho_{2}Q_{2}v_{2}$$

(10) $$F_{\sigma }=\pi d_{o1}\sigma_{12}+\pi d_{o2}\sigma_{23}$$

(11) $$F_{B}=\frac{4}{3}\pi {\left(\frac{d_{drop}}{2}\right)}^{3}\;\rho g$$

where *Q* and *d* represent volume flow rate input and diameter, respectively; subscripts *o* and *drop* denote the outer and the whole droplet. As for the Stokes drag force, we employed the model with the wall effect considered since the diameter of the droplets formed in our device has a similar magnitude as that of the channel [31,40]:

(12) $$F_{D}=3\pi \mu_{3}d_{drop}\left({\bar{v}}_{3}K_{1}-{\bar{v}}_{drop}K_{2}\right)$$

where ${\bar{v}}_{3}$ and ${\bar{v}}_{drop}$ are the mean velocities of the continuous phase and the whole droplet:

(13) $${\bar{v}}_{3}=\frac{4Q_{3}}{\pi d_{channel}^{2}}$$

(14) $$F_{D}=3\pi \mu_{3}d_{drop}\left({\bar{v}}_{3}K_{1}-{\bar{v}}_{drop}K_{2}\right)$$

with $K_{1}$ and $K_{2}$ being the wall effect correction coefficients as:

(15) $$\begin{array}{c} K_{1}=1/[1-2.10443\lambda +2.08877\lambda^{3}-0.94813\lambda^{5}-1.372\lambda^{6}+3.87\lambda^{8}\\ -4.19\lambda^{10}+O\left(\lambda^{11}\right)] \end{array}$$

(16) $$K_{2}=K_{1}\left[1-\frac{2}{3}\lambda -0.1628\lambda^{3}-0.4059\lambda^{7}+0.5236\lambda^{9}+1.51\lambda^{10}+O\left(\lambda^{11}\right)\right]$$

where λ is the dimensionless droplet diameter as follows:

(17) $$\lambda =\frac{d_{drop}}{d_{channel}}$$

Note that Equations (15) and (16) have been proven to show good agreements with the exact solution with λ≤0.8 [31,40]. For larger λ, we may need higher order expansions or the exact solution of Haberman and Sayre [41], which is too long to be expressed here.

By scaling $d_{drop}\sim d_{o2}$, we can obtain the orders of magnitude:

(18) $$F_{D}\sim O\left(10^{-5}\;N\right),\;F_{K}\sim O\left(10^{-9}\;N\right),\;F_{\sigma }\sim O\left(10^{-5}\;N\right),\;F_{B}\sim O\left(10^{-7}\;N\right)$$

Thus, the force balance equation in the current study can be reasonably simplified as:

(19) $$F_{D}=F_{\sigma }$$

Now the diameter of the whole droplet $d_{drop}$ can be obtained by numerically solving the algebraic system above. To estimate the size of the core, we employed [13,34]:

(20) $${\left(\frac{d_{core}}{2}\right)}^{3}=\frac{Q_{1}}{Q_{1}+Q_{2}}{\left(\frac{d_{drop}}{2}\right)}^{3}$$

## 5. Results and Discussion

**Figure 4:**
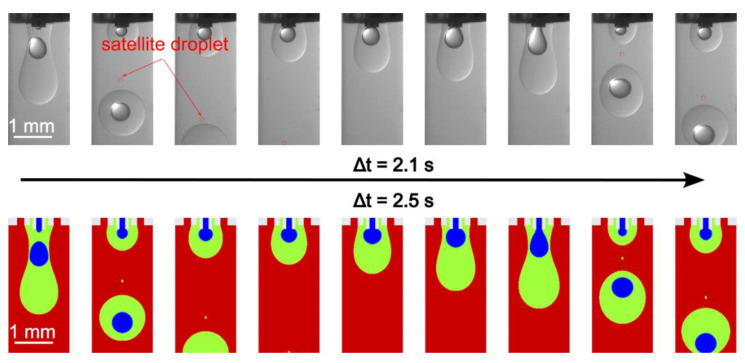
Droplet formation with regular double emulsions at *Q*_1_ = 3 µL/min, *Q*_2_ = 30 µL/min, *Q*_3_ = 250 µL/min.

We first examined the formation process with the continuous phase flow rate *Q*_3_ of 250, 500, and 1000 µL/min, while the inner and middle phase flow rates *Q*_1_ and *Q*_2_ were fixed at 3 µL/min and 30 µL/min, respectively. Figure 4 presents the double emulsion formation in the case of *Q*_1_ = 3 µL/min, *Q*_2_ = 30 µL/min, *Q*_3_ = 250 µL/min over a formation period. The Experimental (top row) and the corresponding simulation results (bottom row) are obtained at nine time steps, with an excellent agreement in the droplet formation pattern. However, the total formation time showed a 16% relative deviation. We observed that the core droplet breaks up before the shell does. A satellite droplet also forms after the break-up of the shell droplet. Over the entire formation process, double emulsions are formed in a regular pattern, with each shell droplet containing a core. In addition, as the whole droplet flows downstream, the core droplet moves downwards quicker than the shell one due to the higher density. This phenomenon is slightly overpredicted in the simulation; see the second image in Figure 4. This might also be caused by the fact that the needles are not precisely tri-axial due to the manufacturing error, evidently by the left skewness in the experimental results.

**Figure 5:**
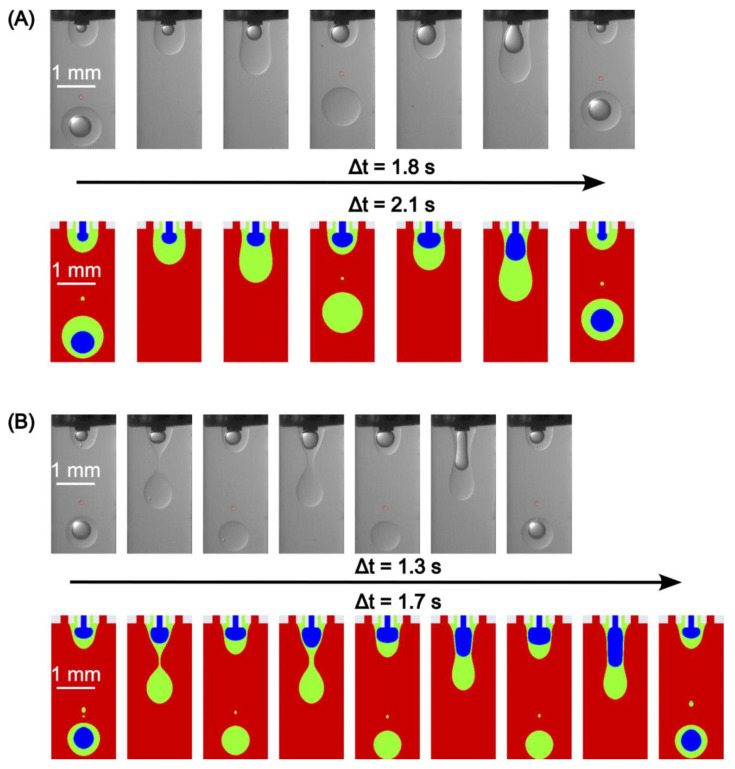
Droplet formation with mismatch at *Q*_1_ = 3 µL/min, *Q*_2_ = 30 µL/min and: (**A**) *Q*_3_ = 500 µL/min; (**B**) *Q*_3_ = 1000 µL/min.

Furthermore, the other two cases with a continuous phase flow rate *Q*_3_ of 500 and 1000 µL/min, while the inner *Q*_1_ and middle *Q*_2_ phase flow rates were fixed at 3 µL/min and 30 µL/min, respectively, Figure 5. Figure 5A shows that with *Q*_3_ = 500 µL/min, double and single emulsions occur alternately, meaning that only one of every two droplets contains a core. Although the time period still shows a 14% deviation between the experiment and simulation, the flow patterns and droplet sizes were captured with remarkable agreement. Worse conditions can be found with *Q*_3_ is 1000 µL/min, Figure 5B, where only one in three droplets contains a core in the experiment, and one in four droplets contains a core in the simulation. Now the flow patterns show a difference within a period. Since it is necessary to avoid the mismatch in applications, the deviation on the mismatched frequencies is not discussed in detail. These unmatched phenomena can be explained by the mismatch of pinch-off locations. The pinch-off location of the middle phase is stretched downwards due to the increased capillary number of the continuous phase, while the pinch-off location of the inner phase remains near the needle tip. Additionally, we also observed the negligible difference between the size of the double emulsion and the single droplet in both experiments and simulations, meaning that the inner phase flow rate only has a slight effect on the whole droplet size. This observation agrees with previous studies [24,26].

**Figure 6:**
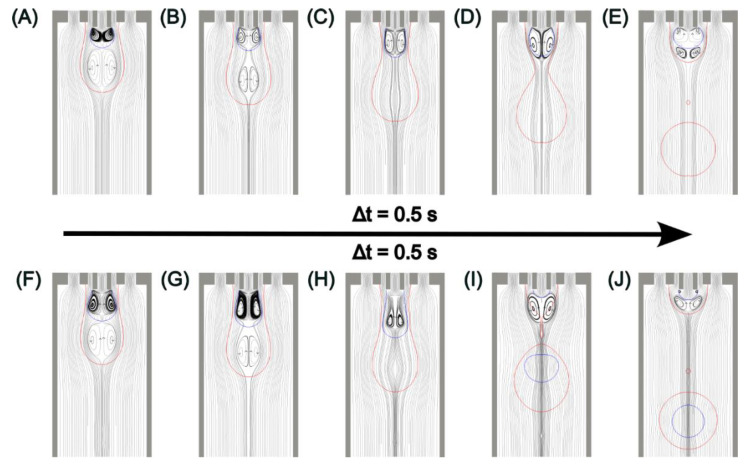
Velocity streamlines near the needle tip at *Q*_1_ = 3 µL/min, *Q*_2_ = 30 µL/min, *Q*_3_ = 500 µL/min: (**A**–**E**) when the mismatch occurs; (**F**–**J**) when the core matches with the shell droplet.

The mismatch phenomenon can be further illustrated along with the velocity streamlines in the case with *Q*_1_ = 3 µL/min, *Q*_2_ = 30 µL/min, *Q*_3_ = 500 µL/min in Figure 6. The blue lines represent the interface between the inner and middle phases, while the red ones stand for the interface between the middle and outer phases. Due to the abrupt expansion of the channel, the velocity profile drastically changes in the vicinity of the needle tips and then develops downstream. Two pairs of vortices, which in fact are two vortex rings due to the axisymmetric geometry, can be observed near the needle tips as a result of the sudden change in the velocity profile, Figure 6A,B. The lower vortex ring formed inside the shell droplet pushes the growing core droplet head backward. The upper vortex ring also acts as an obstacle due to the reverse flow direction along the centreline. As both core and shell droplets grow, Figure 6A–C, the size of the lower vortex ring gradually decreases to null by the shear force of the continuous phase. In contrast, the upper vortex ring increases since the core droplet does not grow beyond the pinch-off location of the shell droplet. After the breakup of the shell droplet, the upper vortex ring crosses the interface between the inner and middle phases, Figure 6D, and is then squeezed by the middle-outer interface and the inner-middle interface. This process forms a new vortex ring from the inner needle tip, Figure 6E. After the mismatch, the growing core droplet accumulated inside the next shell one due to the fixed flow rate input, Figure 6F. Again, as both droplets grow, the size of the lower vortex ring decreases, but the size of the upper one decreases as well in this period, Figure 6F–H. This is because the accumulated growing core droplet is larger, and the vortex ring inside does not attach to the inner needle tip even before the core breaks up. Consequently, the vortex ring is stretched longer and narrower. Thus, more inertia assists the core droplet heading downstream through the space between the vortex ring and the inner-middle interface, Figure 6H. Finally, the core droplet grows beyond the pinch-off location of the shell droplet and breaks up along with the outer emulsion, Figure 6I,J. The whole process then repeats periodically. A similar explanation can be used for the mismatch in the case with *Q*_1_ = 3 µL/min, *Q*_2_ = 30 µL/min, *Q*_3_ = 1000 µL/min. The difference is that the shell droplet pinches off two or three times before the core droplet accumulates to grow beyond the pinch-off position.

To solve the mismatch issue, the core droplet needs to grow beyond the pinch-off location of the shell droplet within each pinch-off period, or $\frac{l_{p2}-\Delta z_{tip1}}{v_{g1}}\leq t_{p2}$. Hence, we define a match ratio of:

(21) $$\xi =\frac{l_{p2}-\Delta z_{tip1}}{v_{g1}t_{p2}}$$

where $\Delta z_{tip1}$ is the inner needle tip elevation difference; $v_{g1}$ is the core droplet growth velocity estimated as:

(22) $$v_{g1}\approx \frac{Q_{1}}{\pi {\left(\frac{d_{o1}}{2}\right)}^{2}}$$

and the pinch-off time of the shell $t_{p2}$ calculated based on the theoretical model as:

(23) $$t_{p2}=\frac{\frac{4}{3}\pi {\left(\frac{d_{drop}}{2}\right)}^{3}}{Q_{1}+Q_{2}}$$

**Figure 7:**
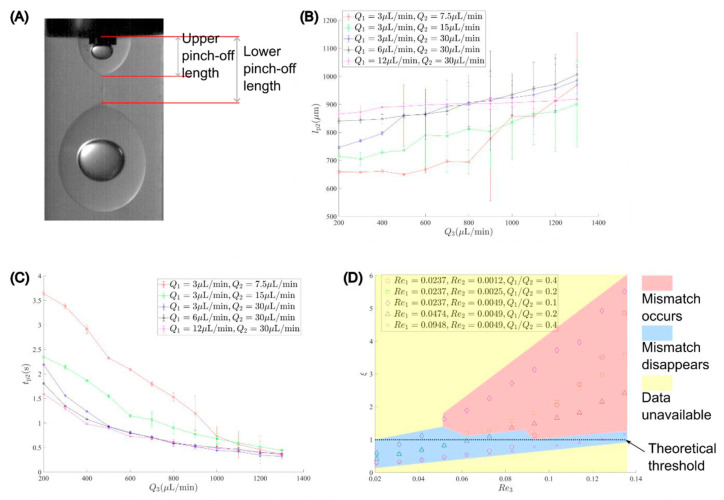
Pinch-off parameters: (**A**) pinch-off length measurement; (**B**) pinch-off length versus the continuous phase flow rate; (**C**) pinch-off time versus the continuous phase flow rate; (**D**) match ratio versus the continuous phase Reynolds number.

However, to our best knowledge, the pinch-off length of the shell droplet, $l_{p2}$, cannot be approximated through a simple model. Thus, we measured it and the pinch-off time experimentally based on the averaged value of the upper and lower pinch-off lengths, Figure 7A. Error bars were calculated from standard deviations of the mean (*n* = 3). We examined *Q*_3_ ranging from 200 to 1200 µL/min with a 100-µL/min interval with the inner phase flow rate *Q*_1_ of 3, 6, 12 µL/min, and the middle phase flow rate *Q*_2_ of 30, 15, 7.5 µL/min, respectively. Note that the corresponding continuous phase Reynolds number, *Re*_3_, and Capillary number, *Ca*_3_, range from 0.02 to 0.14 and from 0.01 to 0.09, respectively. Figure 7B,C show the measured averaged pinch-off length and time against the continuous phase flow rate at different flow rates of the inner and middle phase, respectively. The standard deviations of the mean were calculated from three samples for each case [42].

With increasing *Q*_3,_ the pinch-off length and time of the shell droplet generally increase and decrease, respectively. We also noticed that, when a mismatch occurs, the standard deviation of the pinch-off length drastically increases, while the counterpart of the pinch-off time shows a relatively stable pattern. Additionally, when the sum of *Q*_1_ and *Q*_2_ increases, the pinch-off time for each case decreases and gradually approaches closely as *Q*_3_ increases. In contrast, as the sum of *Q*_1_ and *Q*_2_ increases, the pinch-off length shows a reduction pattern before mismatches occur but a relatively chaotic counterpart due to the mismatches, leading to abrupt increases for different cases. The mismatch issue does not show up in the case of *Q*_1_ = 12 µL/min and *Q*_2_ = 30 µL/min within the *Q*_3_ range in this study, and thus the corresponding line has a negligible change. Equations (21)–(23) indicate that the mismatch issue can be theoretically avoided once the match ratio, ξ, is lower than unity. Increasing $Q_{1}$ or decreasing $Q_{2}$ can reduce ξ. Increasing $\Delta z_{tip1}$ can also decrease ξ, but this option was not examined due to the limitation of the tri-axial needles used in this study. Figure 7D shows the operation map with the match ratio versus the continuous phase Reynolds number for various Reynolds numbers of inner and middle phases. The operation map shows the occurrence of mismatches in experiments. We found a very close threshold at ξ=1, beyond which the mismatch occurs. Additionally, the match ratio generally decreases with an increase in the inner-middle phase flow rate ratio. However, even at the same inner-middle phase flow rate ratio *Q*_1_/*Q*_2_ = 0.4, the one with a higher total flow rate shows a better performance than the other. Hence, it would be better to increase the inner phase flow rate rather than decrease the middle one.

**Figure 8:**
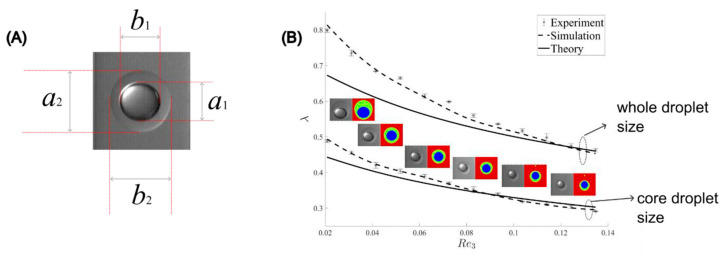
Matched droplets: (**A**) core and shell droplet diameter measurement; (**B**) size distribution comparisons among experiments, simulations, and theory.

After resolving the mismatch issue by increasing the inner phase flow rate, we compared the size distribution among experiments, simulations, and theory. The diameters of the droplets in the experiment and simulation were determined through ImageJ by measuring the vertical and horizontal lengths of at least three whole droplets for each case and then averaged as ${\bar{d}}_{i}=\frac{a_{i}+b_{i}}{2}$, as shown in Figure 8A. Error bars were again calculated from standard deviations of the mean. Figure 8B shows the effect of the continuous phase Reynolds number on the matched droplet size normalized by the channel diameter. As observed, the analytical result on the whole droplet shows a slight deviation from the experimental one when *Re*_3_ ranges from 0.02 to 0.06 and asymptotically approaches good agreement with *Re*_3_ increasing from 0.06 to 0.14. The analytical result of the core droplet diameter shows a better prediction against the experimental data within the range of *Re*_3_ in our current study. This can be explained by the approximated wall effect correction model since increasing *Re*_3_ decreases the dimensionless droplet diameter λ, leading to less influence of the wall effect; see Equations (15) and (16). Furthermore, the reduction in the whole droplet diameter also reduces the deformation effect, which results in a better agreement with the assumption of a spherical droplet. In contrast, the CFD simulation results generally overlap with experimental data, indicating that the current numerical model is valid to capture the double emulsions in the dripping regime through the tri-axial capillary device.

## 6. Conclusions

We experimentally, numerically, and theoretically investigated the formation process of double emulsion in a vertically arranged tri-axial co-flow capillary device. The volume of fluid (VOF) method and continuous surface force (CSF) model were used for the numerical simulation to catch the details of fluid flow. An analytical model based on a simple force balance was built to estimate the droplet size distribution in accordance with the continuous phase Reynolds number. We found that mismatches of core and shell droplets under certain flow conditions can be captured both experimentally and numerically. To quantify the mismatch, we proposed a semi-theoretical model by matching the pinch-off length of the shell droplets with the product of the growth rate of the core and the pinch-off time of the shell. The mismatch issue is expected to be avoided if the match ratio is lower than unity, which was validated with experimental data. Regarding the reduction in the match ratio, we found that increasing the inner phase flow rate shows better performance than reducing the middle one. Considering the wall effect, the analytical model for predicting the size of matched double emulsions showed slight deviations from experiments if the continuous phase Reynolds number is lower than 0.06. However, the behaviour asymptotically approaches good agreement in the Reynolds number range of 0.06 to 0.14. The numerical simulation generally agreed with the experimental data under the investigated flow conditions.

## Figures and Tables

**Table 1:** The dimensions of the tri-axial needles.

Needle/Phase	Inner Diameter (µm)	Outer Diameter (µm)	Thickness (µm)
1	178	356	89
2	508	813	152
3	1190	1650	229

## Data Availability

The data presented in this study are available on request from the corresponding author.
